# The impact of phosphate scarcity on pharmaceutical protein production in *S. cerevisiae*: linking transcriptomic insights to phenotypic responses

**DOI:** 10.1186/1475-2859-10-104

**Published:** 2011-12-07

**Authors:** Ali Kazemi Seresht, Eva Akke Palmqvist, Lisbeth Olsson

**Affiliations:** 1 Protein Expression, Novo Nordisk A/S, Novo Nordisk Park 1, 2760-Måløv, Denmark; 2 Industrial Biotechnology, Department of Chemical and Biological Engineering, Chalmers University of Technology, 41296-Gothenburg, Sweden

**Keywords:** Phosphate regulation, heterologous protein production, chemostat cultivations, human insulin, secretory flux, *TPI1 *promoter

## Abstract

**Background:**

The adaptation of unicellular organisms like *Saccharomyces cerevisiae *to alternating nutrient availability is of great fundamental and applied interest, as understanding how eukaryotic cells respond to variations in their nutrient supply has implications spanning from physiological insights to biotechnological applications.

**Results:**

The impact of a step-wise restricted supply of phosphate on the physiological state of *S. cerevisiae *cells producing human Insulin was studied. The focus was to determine the changes within the global gene expression of cells being cultured to an industrially relevant high cell density of 33 g/l cell dry weight and under six distinct phosphate concentrations, ranging from 33 mM (unlimited) to 2.6 mM (limited). An increased flux through the secretory pathway, being induced by the *PHO *circuit during low P_i _supplementation, proved to enhance the secretory production of the heterologous protein. The re-distribution of the carbon flux from biomass formation towards increased glycerol production under low phosphate led to increased transcript levels of the insulin gene, which was under the regulation of the *TPI1 *promoter.

**Conclusions:**

Our study underlines the dynamic character of adaptive responses of cells towards a change in their nutrient access. The gradual decrease of the phosphate supply resulted in a step-wise modulated phenotypic response, thereby alternating the specific productivity and the secretory flux. Our work emphasizes the importance of reduced phosphate supply for improved secretory production of heterologous proteins.

## Background

The long traditional attention and thus gathered familiarity with *Saccharomyces cerevisiae *is grounded on the deep knowledge about its genetics, physiology and cultivation techniques, making this eukaryote the main workhorse to study essential biological phenomena. Clearly, the gain of such immense knowledge fruited in many commercial success stories, when the formerly underrated baker's or brewer's yeast matured to one of the most widely used hosts for a large portfolio of products derived by means of recombinant DNA technology. Regarding its revenue and market, the production of active pharmaceutical ingredients (APIs) represents currently with annual global sales of approx. US$ 100 billion (reviewed by [1]), a significant field of research for improved yeast cell factory design.

The biotechnological production of human Insulin in *S. cerevisiae *(comprehensively reviewed by [2]) is considered to be the first of such successful commercial achievements, and represents even today, due to its enormous medical and market value, a highly important field of research. Despite the recent developments within the field of metabolic engineering and synthetic biology, which mostly target the production of metabolites like organic acids [3] and the reinforced alternatives to former petrochemical-based compounds (reviewed by [4] and [5]), only little novel engineering has been achieved in yeast with respect to its secretory abilities. Strain engineering approaches towards enhanced secretory production of APIs remain essentially on the levels of target gene amplification, over-expression of few ER-associated foldases [6], and the engineering of modified secretion signals that supposedly aim at optimizing the trafficking and release of the heterologous package (reviewed by [7]). The secretory machinery of eukaryotes like *S. cerevisiae*, embodying its severe quality control abilities and the performance of complex posttranslational modifications, is still providing a partly undiscovered and thus fruitful ground for biotechnological progress with respect to both quantitatively and qualitatively enhanced production of APIs. In particular, the popular metabolic engineering philosophy of channeling an increased flux towards a given metabolic pathway turns out to be more challenging when it comes to increasing the secretory flux of the cells, as engineering of yeast protein factories still remains on the level of chaperone and ER resident folding catalysts (reviewed by [8])

One of the key elements for optimized production of APIs has traditionally been the design and deployment of growth media in which the cells are provided with excess nutrients to grow, multiply, and to produce the protein of interest. Such nutritious media support first of all the growth of the recombinant cells, compensating the additional burden derived from the over-production of the heterologous protein. But, could the excess supply of nutrients potentially saturate, and thus partly silence, biosynthetic and reshuffling processes of key API precursors? And would these processes, when stimulated under nutrient hunger, improve the processing, e.g. maturation and secretion of the protein of interest?

Growth under nutrient limited conditions has been investigated in many cases during the past, facilitating advanced physiological characterization of the cells. We learned that by throttling the evolutionary favored biomass formation, the salvaged energy and metabolic precursors can be re-distributed towards a particular biosynthetic process, leading to the development of novel bioprocessing strategies based on limited nutrient supply, e.g. fed batch and continuous cultivations. As a matter of fact, the yeast *S. cerevisiae *has great capabilities of responding to alternations in its nutrient availability. As a unicellular system, it has evolutionary matured measures to quickly respond to any changes in its environment, and it does so through re-distribution of energy and biosynthetic fluxes.

Inorganic phosphate (P_i_) is an essential nutrient for any organism, as it is required for the biosynthesis of nucleotides, phospholipids and metabolites, in energy metabolism, and it acts as an important messenger in signal transduction processes. Sophisticated and efficient regulatory circuits are needed for proper management of mechanisms like acquisition, storage, and utilization of phosphate. Recent studies have examined the impact of limiting nutrient availability, e.g. C-, N-, P-, and S-limitation, on a global scale of endo-metabolome [9], proteome [10], transcriptome [11], or in combination [12], to name a few. These studies (and references herein) revealed close interconnection among the global transcriptome and the specific growth rate, and further highlighted the major physiological impact of carbon limitation compared to the limitation of other nutrients, e.g. nitrogen, phosphor and sulfur.

Yet, we suspect that the adaptive response of cells towards a change in their nutrient access is a dynamic process, meaning that cellular regulatory events do not necessarily happen at the point of any particular limitation, but follow a more transient progression from one physiological bottleneck to the other. The understanding of such regulatory events necessitates, besides to the perception of physiological changes at limiting levels of a given nutrient, also the observation of the progressive changes during a step-wise reduced supply of that particular nutrient. In this study, we followed the transcriptional impact of a step-wise reduced supply of phosphate to cells that were growing under carbon limited condition in chemostat cultivations. As all our investigated *S. cerevisiae *cultures were growing with essentially the same fixed specific growth rate, i.e. being fed by a controlled and limited supply of carbon, any cellular response being not related to the alternating phosphate supply was out-normalized. Besides, we put, to our knowledge for the first time, the impact of a variable phosphate supply in a context of the production of a heterologous protein, in our case a human Insulin Analogue Precursor (IAP), in *S. cerevisiae*. Yet another novelty of our work is the fact that we examined the cells in a more industrially relevant context than it is generally done in nutrient limitation studies. The cultivations of the cells were performed at high cell density in sophisticated continuous chemostat cultivations and the analysis were carried out under steady state conditions, increasing the quality of the obtained biological insights. The *S. cerevisiae *cultures in this study grew at 10 fold higher glucose levels in the feed supply as compared to standard laboratory practice, resulting in reduced P/C ratios (phosphate to glucose ratio) and thus lower residual phosphate levels.

To address the impact of low P_i _on the physiological state of *S. cerevisiae *in a more progressive manner, we carried out individual cultivations at six distinct phosphate supplies, chosen to cover the range from excess to limiting levels. We monitored general physiological changes, in terms of biomass formation, major exo-metabolite secretion, and insulin production, in relation to a diminishing phosphate supply. We followed the global transcriptome as a function of supplied P_i _in two complementary ways: in a rigid way, based on a differential expression analysis (DEA) approach among cultures growing under high versus low P_i _conditions, and in a consecutive way, based on a significant profile analysis (SPA) approach following the progression of transcriptional changes from carbon limited to phosphate limited growth. Our data contribute to the fundamental understanding of cellular responses that are induced when *S. cerevisiae *cells sense a diminishing phosphate supply. In addition, we emphasize the importance of both the design of growth media with reduced phosphate levels and the choice of promoter for the expression of the recombinant gene, resulting in improved production of the heterologous protein of choice.

## Results

**Figure 1:**
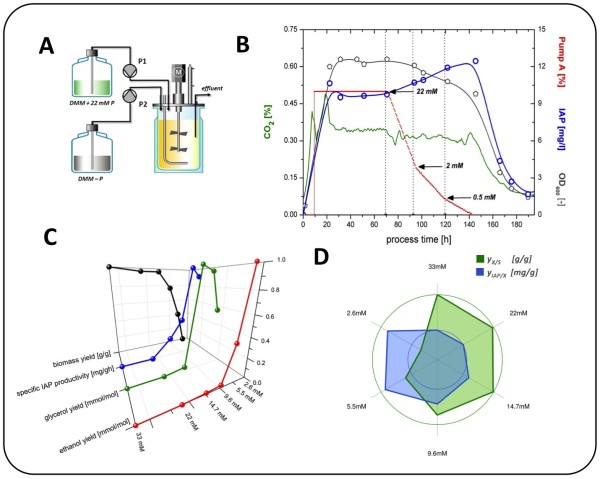
**Physiological impact of reduced phosphate supply in chemostat cultivations.A**. Schematic set-up of the P_i _wash-out experiment. The gradual decrease of the feed supply rate (pump P1) was compensated by a gradual increase of the feed rate of a medium lacking any phosphate source (DMM-P, pump P2). The overall dilution rate was thus kept constant, and the residual phosphate in the culture was washed out. **B**. An overview on the impact of decreasing phosphate supply during the wash-out process. The insulin titers (IAP, blue circles) increased when the phosphate supply was restricted (pump A, red dashed line). **C**. representation of the biomass yield (black, 0.13-0.43 g/g), IAP productivity (blue, 0.196-0.392 mg/gh), glycerol yield (green, 0-0.615 mmol/mol), and ethanol yield (red, 0-12.68 mmol/mol), as a function of the supplied phosphate level in individual cultures. All four parameters were normalized to a range of zero (min. values) to one (max. values). **D**. radar plot representation of the biomass yield (green) and the corresponding IAP yield (blue).

Initially, we investigated the impact of a reduced phosphate supply on the metabolism and, more importantly, on the IAP productivity. The aim was to identify a physiologically feasible range of the supplied phosphate (P_i_) level. Based on the outcomes, a range of interest was identified, and a comprehensive study on the global impact of sequentially decreasing P_i _was designed. For identifying the impact of reduced P_i _supply on IAP production, we chose to gradually wash-out the P_i _from the initial medium whilst maintaining the specific growth rate of the cells, using a fixed dilution rate in the chemostat set-up. The gradual decrease of the feed supply rate (pump 1) was compensated by a gradual increase of the feed rate of a medium lacking any phosphate source (pump 2), thereby maintaining the overall feed rate constant and equal to the effluent flow (Figure 1A). The P_i _wash-out was initialized after keeping the culture for a minimum of five volume exchanges at steady state conditions. The estimated P_i _levels in the culture were calculated, neglecting possible delays due to mixing time, as a relatively high stirrer speed was chosen (1000 rpm). Before reaching the point of cell wash-out (absolute P_i _limitation) in the later stage of the process (after 200 h of cultivation, data not shown), a clear impact of the reduced P_i _supply to the IAP production and biomass formation was found (Figure 1B). An enhanced IAP production was observed at reduced P_i _levels, although the overall metabolism, displayed by the CO_2 _profile, seemed to be only slightly affected.

### Physiological characterization of the cells with respect to the impact of P_i _supply

To understand the cause of increased IAP production on a more ample perspective, we ran six separate chemostat cultivations, supplying six distinct P_i _concentrations 33 mM, 22 mM, 14.7 mM, 9.6 mM, 5.5 mM, and 2.6 mM, respectively, thus covering the determined range of interest derived from our initial experiment. All six cultivations were analyzed for general exo-metabolites, i.e. residual glucose, ethanol, glycerol and acetate, as well as for the secreted amount of the target protein (IAP), biomass (via OD_600 _and cell dry weight analysis), and global gene expression levels (on oligonucleotide microarrays). The respiratory activity of the cells (based on the carbon dioxide production level) was monitored on-line and used for steady state determination. Once the cultures fulfilled and maintained steady state conditions for 10 generations, a sampling regime of two samples per day was followed for 4-5 days. The resulting samples were analyzed as described, and the corresponding yields on biomass (production yields, y_i/X_) and glucose (consumption yields, Y_i/S_) were determined (Figure 1C).

A first glance on the physiological observations pictured major changes in the metabolism and IAP expression within the chosen range of phosphate supply. The biomass formation yield (y_X/S_) did not show any significant changes within the P_i _range of 33 mM (0.424 ± 0.008 g/g) to 14.7 mM (0.421 ± 0.004 g/g). Also, no significant changes in the glycerol and ethanol yields were detected in this range (changes within the standard deviation). Yet, a first increase of the IAP production yield (y_P/X_) was noticeable, increasing 22.6%, from 1.93 ± 0.08 mg/g (33 mM P_i_) to 2.37 ± 0.04 mg/g (14.7 mM P_i_). When the supplied P_i _level was further decreased to 9.6 mM, major metabolic shifts were observed, leading to a re-distribution of the carbon flow from biomass formation (Y_X/S _decreased about 14.6% to 0.362 ± 0.004 g/g glucose) towards both glycerol formation (Y_G/S _= 0.615 ± 0.041 mM/M glucose) and ethanol formation (Y_E/S _= 0.287 ± 0.031 mM/M glucose), indicating a shift from an initially respiratory to an appearing respiro-fermentative metabolism. The IAP production yield further increased to y_P/X _= 2.98 ± 0.09 mg/g and was thus 54% higher than the initial yield at 33 mM P_i_. The further decrease of the P_i _supply led to a major loss of biomass yield, due to augmented by-product formation (Figure 1D). A new range of interest with respect to the following transcriptome analysis was chosen, thus being further narrowed down to the range of 22 mM to 9.6 mM phosphate supply, as this range showed an interesting increase of IAP productivity without significant loss of carbon flux towards by-product formation.

### Transcriptome analysis I: Differential Expression Analysis (DEA) of [22 mM P_i_] vs. [9.6 mM P_i_]

**Figure 2:**
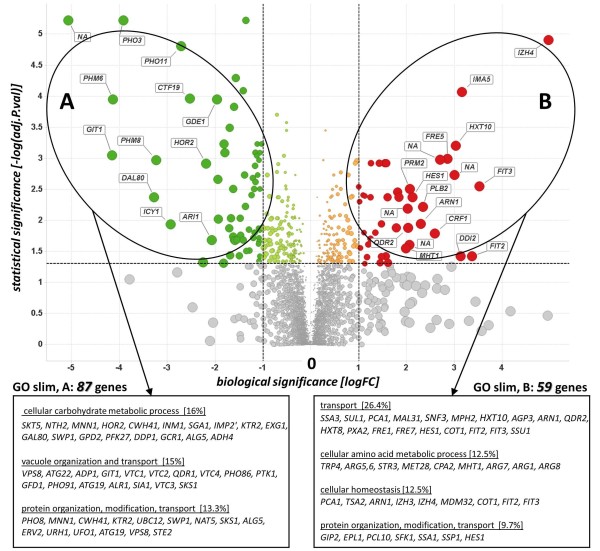
**A volcano plot representation of the differentially expressed genes in a pair-wise comparison of cultures growing at high phosphate level [22 mM] to low phosphate level [9.6 mM]**. The significance cut-off was set to a FDR of 0.05 (-log(adj.P.val ≥ 1.3), the biological cut-off was set to a fold change of ± 2 fold (-1 ≥ logFC ≥ 1), the top 30 differentially expressed genes are labeled with their corresponding gene ID, the absolute fold change of all genes are represented by the circle size, the five different color codes used represent insignificant genes (grey), both biologically and statistically significant genes being up-regulated (red, B) and down-regulated (green, A) at [22 mM] P_i_, statistically but not biologically significant genes being up-regulated (orange) and down-regulated (light green) at [22 mM] P_i_, respectively. A GO slim enrichment analysis of the gene sets A and B revealed the over-representation of the pictured GO terms within the gene sets A and B (%), the group of genes with an unknown biological function for gene set A (26%) and gene set B (25%) was excluded from further analysis.

We carried out a pair-wise comparison between the cultures grown at 22 mM and 9.6 mM phosphate for exploring the global transcriptional impact on cells under low and high phosphate levels, respectively. We identified 381 genes being significantly differentially expressed (statistical significance, Benjamini & Hochberg FDR adjusted *p *≤ 0.05) in a comparison of cultures supplied with 9.6 mM phosphate [9.6 mM] to the control culture, being supplied with 22 mM phosphate [22 mM]. The majority of these genes (217) were up-regulated in [9.6 mM]. A second significance trap (biological significance) was set to further narrow down the genes of interest, choosing a fold change cut-off of abs(FC) ≥ 2, resulting in a decrease of significant differentially expressed genes to 146 genes, of which 87 were up-regulated in [9.6 mM]. Within the top ten genes being up-regulated, we identified five phosphatases *PHO3 *(15.1-fold), *PHM6 and PHM8 *(17.6-fold and 9.4-fold), *PHO11 *(6.5-fold), and *HOR2 *(4.6-fold), besides to the permease *GIT1 *(17.8-fold) being highly expressed under low P_i _conditions. AGene Ontology (GO Slim) enrichment analysis was performed and enriched categories (Figure 2), which together represented the majority of the identified genes being either up- or down-regulated, were identified. The enriched categories related to vacuole re-organization and protein maturation and transport were considered to be very interesting, as the secretory production of the heterologous IAP was expected to be positively affected by the up-regulation of the genes involved in the secretory machinery and the recycling compartment of the cells, presumably leading to an increased protein processing capacity of the cells. The third enriched category of carbohydrate metabolism revealed a first indication that a cross-talk between phosphate supply and glycolytic flux does exist. As the recombinant insulin gene cassette was put under the regulation of the constitutive *TPI1 *promoter, we assumed another, possibly positive, effect of low P_i _supply to IAP expression due to the choice of a glycolytic promoter.

One drawback of such a stringent Differential Expression Analysis (DEA) approach is the risk of losing potentially valuable information on co-regulated genes and their inter-connection among distinct pathways. By using statistical and biological cut-offs for separating the wheat from the chaff in a biological sense, only pieces of a big regulatory picture are revealed, mostly due to the fact that different steps in a given metabolic pathway can be more or less transcriptionally regulated, depending on the complexity of the encoding enzymes. As shown in (Figure 2), the sixth part in the lower right part of the plot-representing genes which are highly up-regulated in the high P_i _supplied culture [22 mM], yet which failed to pass the chosen statistical trap-encloses significantly more genes as compared to the top right sixth part. Also, the chosen biological cut-off led to a non-observance of 65% of the statistically significant genes (top middle sixth part), which potentially would contribute to a more sophisticated biological mining of the observed physiological state of the cells. This possible drawback was counteracted in a second approach, where the focus was set to genes with a significant expression profile as a function of supplied phosphate.

### Additional insights to transcriptional changes via the Significant Profile Analysis (SPA) approach

**Figure 3:**
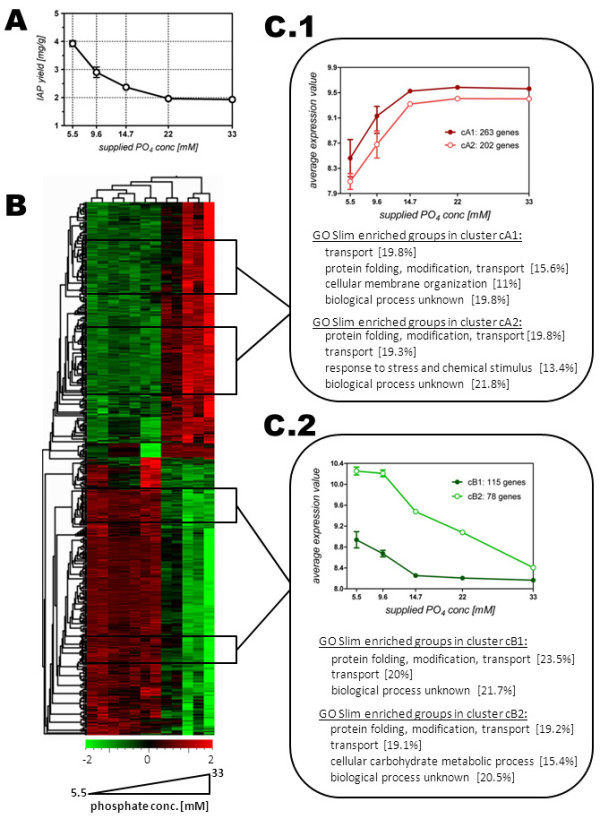
**Identification of significant gene expression profiles in relation to the observed IAP expression dynamics. A**. The insulin yield on biomass, as a function of supplied phosphate.** B**. a heat map of the hierarchical clustering analysis based on genes picturing a significant expression profile as a function of supplied phosphate; the lowest examined phosphate level [2.6 mM] was excluded from the SPA analysis; the color code represents the scaled expression values. **C.1**. Two clusters cA1 (263 genes) and cA2 (202 genes) were selected, harboring genes with declining expression profile with diminishing phosphate levels. The GO slim enrichment analysis identified each four categories, covering 66% (cA1) and 74% (cA2) of genes within each cluster.**C.2**. Two clusters cB1 (115 genes) and cB2 (78 genes) were selected, harboring genes that showed, similarly to the IAP yield, an increasing expression profile with decreasing phosphate levels. The GO slim enrichment analysis identified three (cB1) and four (cB2) enriched categories, covering 65% (cB1) and 74% (cB2) of gene within each cluster, respectively.

Our intention then was to salvage more in depth insights into the impact of the altered phosphate supply and its relation to insulin production. We suspected that, by considering the expression pattern of any significant gene, and by identifying gene clusters which show a progressive trend as a function of the change in their phosphate availability, more in depth biological insights regarding regulatory events can be found. For this, we used a regression based methodology which is in more common practice in time course experiments [13]. By substituting the time factor with our compiled range of interest for P_i _concentration, the examined phosphate levels were taken into account in a progressive manner. However, we did not include data of the culture which was supplied with the lowest P_i _level [2.6 mM], as we considered the physiological changes that occurred at this level to be too abundant and not in favor of the production of our target protein. Using the MicroArray Significant Profiles (maSigPro) tool, 1289 genes were identified which pictured a significant alternating expression profile as a function of the supplied phosphate amount. These profiles were then separated in an array of nine clusters and further sieved for similarity with the more macroscopic determined trend of y_P/X _(Figure 3A). We identified each two clusters of genes with increasing (cB1 and cB2) and two clusters with decreasing (cA1 and cA2) trend as a response to decreasing P_i _supply.

The clusters cA1 and cA2 showed a general similarity, and more interestingly, no significant change in the expression for the range of 33 mM to 14.7 mM P_i_, reflecting our previous observations with respect to the general physiological state of the cells within this range. As compared to the DEA results, and taking into account the quantity of genes found in cA1 (283 genes) and cA2 (202 genes) picturing a significant profile, additional information on down-regulated biological processes were found with respect to a decreasing phosphate supply. The clusters cB1 (115 genes) and cB2 (78 genes) constitute genes which showed an increasing expression profile as a response of decreasing P_i _supply. Again, cluster cB1 shows an interestingly high similarity to our physiological data, as it represents genes with increased transcript levels when the phosphate concentration is reduced to 9.6 mM and below. In contrast, the genes encompassed within cluster cB2 pictured a more sensitive response to the reduction of phosphate in the corresponding cultures, as their expression changes throughout the entire range of examined P_i _supply. All four clusters where then surveyed for enriched GO Slim categories, (Figure 3C.1 and 3C.2), representing the majority (> 65%) of the comprising genes of the individual clusters. Besides to the significantly enriched group of gene with unknown biological process (> 20%) which was excluded from the data mining, we identified GOs related to protein maturation and transport being enriched in all clusters, representing approx. half of all clustered genes (see also additional file 1). As both increasing and decreasing expression profiles showed enriched GOs in these terms, we regarded these outcomes as a clear indication of a changing flux through the cell-internal secretory pathway, and with respect to the observed boost of the secretory production of the heterologous protein IAP, as highly valuable. Interestingly, the cluster of genes with the most sensitive response towards the variation of the P_i _supply (cB2) was also enriched in the GO categories related to carbohydrate metabolism. Considering the transcriptional regulation of the insulin gene, which is under the regulation of the glycolytic *TPI1 *promoter, this finding implies a possible increase of the IAP transcript levels to be a cause for its increased production under reduced phosphate conditions.

**Figure 4:**
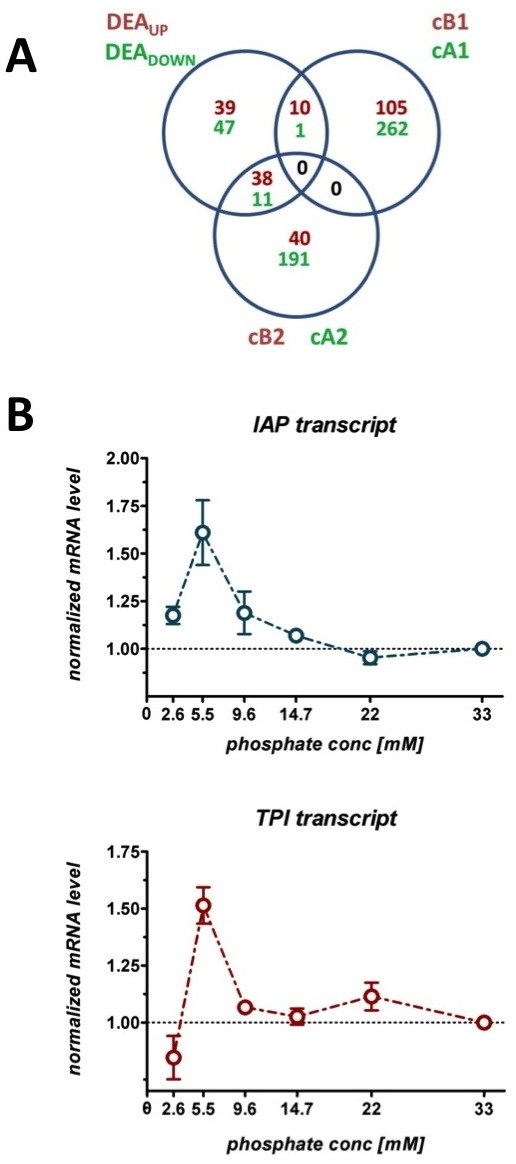
**Mining the impact of phosphate scarcity by using complementary gene expression data sets. A**. Venn diagram representing the overlapping genes identified with the DEA approach (comparison of cultures growing at low and high phosphate levels) and the SPA approach (consecutive analysis of gene expression patterns as a function of phosphate supply; gene numbers with increasing expression at low phosphate (dark red) and gene with decreasing expression at low phosphate (green) are highlighted. **B**. RT-PCR analysis of the transcript levels of insulin gene (blue) and the *TPI1 *gene (red) as a function of supplied phosphate; increased levels of the IAP transcript were measured under low phosphate levels; the mRNA levels are normalized to their initial quantities at high phosphate levels [33 mM]; cultures grown under limited phosphate supply [2.6 mM] showed decreased *IAP *and *TPI1 *transcript levels.

In this study, additive information on transcriptional changes was obtained when two different methodologies were applied. Considering the consecutive expression pattern of genes over the full range of the examined phosphate levels proved to enrich the data derived from a transient analysis of the transcriptional changes among low (< 9.6 mM) versus high (> 22 mM) P_i _levels. As shown in (Figure 4A), both the pair-wise comparison (DEA approach) and the regression based profile analysis (SPA approach) led to a complementary amount of significant genes which supported the process of biological mining of the observed phenomena. Both, the overlaps between down-regulated genes in the DEA approach and the SPA derived clusters cA1 (1) and cA2 (11), and the up-regulated genes among the DEA approach and the clusters cB1 (10) and cB2 (38), respectively, represent the complementation of both methodologies.

### The impact of reduced phosphate supply on the transcription of the insulin gene

For investigating the directed impact of the reduced P_i _supply to the target gene expression, we performed RT-PCR experiments on both the transcripts of the insulin gene (*IAP) *and for the *TPI1 *gene *(TPI)*, as the regulation of *TPI1 *was expected to have an impact on the expression levels of insulin (same promoter). As suggested by the gene ontology enrichment analysis, an increased glycolytic activity was observed when cultures were grown under low phosphate conditions [below 14.7 mM]. Although we identified relatively distinct parts of carbohydrate metabolism to be differentially regulated, i.e. glycerol biosynthesis (*GPD2, HOR2) *and the down-stream part of glucose fermentation (*PYK2, PDC6, ADH1,4*), we predicted the central node of these two pathway branches within the glycolysis, encoded by *TPI1*, to be affected in accordance.

The obtained transcript levels of the *IAP *and *TPI1 *were normalized to the levels found under high (33 mM) phosphate levels (Figure 4B). Both genes showed a similar profile and significantly increased levels under low P_i_. In addition to our first conjecture that the increased flux through the secretory pathways of the cells benefits the heterologous insulin production, we concluded that the elevated insulin production yields observed under low phosphate are also enrooted on its increased transcript levels.

## Discussion

Under phosphate limiting conditions, the phosphate signal transduction (*PHO*) pathway is activated, leading to a coordinated cellular response through a transcriptional induction of a wide regulatory circuit, involving genes coding for a high affinity transport system (*PHO84, PHO89)*, acid phosphatases (*PHO5, PHO11, PHO12)*, alkaline phosphatase (*PHO8)*, polyphosphate storage (*PHM1 to PHM5)*, and genes involved in the catabolism of alternative phosphorous sources (*GIT1, GDE1, HOR2) *[14-16]. The actual activation of the *PHO *pathway is triggered by the inhibition of the cyclin-dependent kinase (CDK) complex Pho80-Pho85 via the CDK-inhibitor Pho81 [17]. Very recently, it has been shown that a small molecule ligand, inositol heptakisphosphate (IP_7_), is required for inducing the inhibitory activation of Pho81 on the CDK complex [18], thereby preventing that the kinase complex would hyperphosphorylate the transcription factor Pho4. The unphosphorylated Pho4 can then associate with the nuclear importer receptor Pse1 and translocate into the nucleus, and in cooperation with another transcription factor Pho2, activate the *PHO*-responsive genes [19].

**Figure 5:**
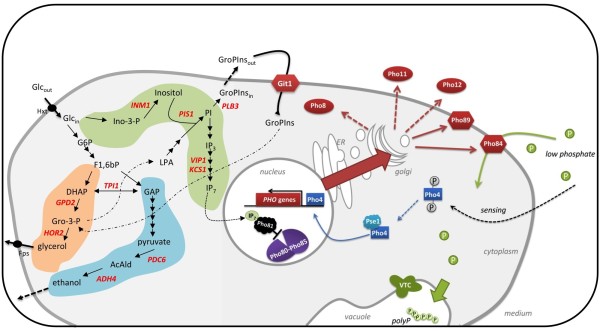
**A schematic view on the inter-connection of cellular responses during growth on low phosphate levels**. All labeled genes (left hand side) and illustrated proteins (right hand side) showed significantly up-regulated transcript levels when cells were grown under low phosphate levels [9.6 mM]. The up-regulated pathways included the glycerol biosynthesis (orange background), the lower part of glucose fermentation, being significantly increased downstream from the pyruvate decarboxylation step (blue background), the phosphatidylinositol biosynthesis and the lower part of inositol phosphate biosynthesis pathways (green background), yielding in heptakisphosphate (IP_7_) which has been shown to regulate the inhibitory activity of Pho81, thus allowing the transcription factor Pho4 to access the nucleus and induce the *PHO *pathway. Metabolites: Glc glucose, G6P glucose-6-phosphate, F1, 2 bP fructose-1,2-bisphosphate, DHAP dihydroxyacetone, Gro-3-P glycerol-3-phosphate, GAP glyceraldehyde-3-phosphate, AcAld acetaldehyde, Ino-3-P inositol-3-phosphate, PI phosphatidylinositol, IP_3 _inositol triphosphate, IP_7 _inositol heptakisphosphate, GroPIns glycerophosphoinositol, LPA lyso-phosphatidic acid.

Our data show a significant up-regulation of all named high affinity phosphate transporters and acid phosphatases but one, being *PHO5 *(Figure 5, top right). It has been shown that enzymes involved in inositol pyrophosphate synthesis appear to be essential for the regulation of *PHO5 *expression [20]. In fact, *KCS1*, which has been shown to suppress *PHO5 *expression under low phosphate conditions, is in this study more than three fold up-regulated. Together with the two up-regulated genes *INM1 *and *PIS1 *which are both encoding enzymes further upstream in the inositol phosphate pathway, we have an indication for the cross-talk of inositol pyrophosphate formation and *PHO *pathway activation. Evidence of the cross-talk has also been found by [21], who reported the cellular component stimulating the Pho81-dependent inhibition of the CDK complex to be the product of both IP_6 _kinases Kcs1 and Vip1. The implications of the non-differentially expression of *PHO5 *under reduced phosphate concentrations for the design of improved expression systems is significant. The *PHO5 *promoter has been a preferred regulator of heterologous protein production, as cells which have been grown under phosphate limitation over-express this acid phosphatase gene and consequently the heterologous protein that has been put under the inducible *PHO5 *promoter [22,23]. Our data underlines the benefit of using the non-inducible *TPI1 *promoter, as the expression of the target protein was positively affected by the reduction of the phosphate level in the media through-out the examined phosphate range, whereas the a *PHO5*-dependent expression would most likely show a benefit only at limiting but not reduced phosphate levels.

Interestingly, when the phosphate concentration in the medium is decreased, polyphosphate accumulation in the vacuoles mediated by the vacuole transporter chaperone (VTC) complex, of which the four sub-units are encoded by *PHM1 *to *PHM4 *(alias *VTC1-4*), was 2-3 fold up-regulated (Figure 5, lower right). Although this seems to be contradictory, as polyphosphates represent a P_i _reserve used during periods of P_i _starvation [24], the increased intracellular phosphate levels, mostly obtained by the high-affinity phosphate uptake mediated by Pho84, need to be stored as polyphosphates in the vacuoles for avoiding possible negative feedbacks to the uptake system. As reported by Ogawa and co-workers [16], increased cell-internal P_i _levels would lead to inhibitory effects on Pho84, thus counter-acting the high-affinity transport system.

Our data show that the inositol biosynthesis pathway is partly up-regulated with decreasing phosphate concentration in the medium. As the inositol concentration in the media was kept constant for all investigated media formulations, and *INO1*, encoding the first and rate-limiting enzyme in inositol biosynthesis, did not show any significant differential expression, no indication of inositol limitation were found. Still, with the up-regulation of *INM1 *and *PIS1*, we had indications that the formation of phosphatidylinositol (PI) was increased (Figure 5, green cloud). Being an essential lipid for eukaryotic cells, PI acts as a precursor for sphingolipids, polyphosphoinositides, and most interestingly for glycerophosphoinositol (GroPIns). The latter is derived trough deacylation via phospholipases like *PLB2*, which in turn was also up-regulated. It has been shown that GroPIns, as a major PI catabolite in inositol containing medium, is released to the extra-cellular milieu [25]. Under both phosphate and inositol limiting conditions, GroPIns can be transported into the cells, mediated by a permease encoded by the *GIT1 *gene [26]. This permease gene was found to be more than 17 fold increased and among the top five genes we identified in this study to be significantly differentially expressed in the pair-wise comparison of high vs. low P_i_. Our data underline the outcomes of [27] who have reported an accumulation of *GIT1 *transcripts when phosphate limitation is artificially imposed in a Δ*pho85 *strain. The findings are also in accordance with Almaguer et al. [28] who also have reported that *GIT1 *transcript levels and Git1p activity are higher in cells starved for phosphate with or without inositol limitation. The latter study also concludes that GroPIns can act as the sole phosphate source for cells.

Both the physiological and the transcriptional changes observed in this study suggest a carbon flux re-distribution within the glycolysis towards increased glycerol formation. The increased transcript levels of *GPD2*, a NAD-dependent glycerol-3-P dehydrogenase, and *HOR2*, a glycerol-3-P phosphatase, (Figure 5, orange cloud) lead to a more than 30 fold increase of the extracellular glycerol (Figure 1C). Interestingly, *GPD2 *and *HOR2 are *known to be induced under anaerobic conditions and hyperosmotic stress, respectively [29], yet these are not the conditions of our study. However, it has been reported that the *GPD2 *transcript levels respond to fluctuations of the cytosolic NADH/NAD^+ ^ratio and thus to the redox state of the cytosol [30]. In a recent study committed by Lu et al. [31], it has been shown that components of the *PHO *pathway play important roles in NAD^+ ^metabolism. It has been reported that low P_i _conditions cause increased intracellular nicotinamide riboside (NmR) levels, which are derived through the catalysis of nicotinamid nucleotids (NMR) by the alkaline phosphatase Pho8, leading to a release of phosphate. We found that the transcript of *URH1*, a key enzyme in the NmR-derived NAD^+ ^biosynthesis, is increased with decreasing P_i _supply. Taken together with the increased flux through the NAD-requiring glycerol biosynthesis, these outcomes underline the cross-talk between phosphate signaling and NAD^+ ^metabolism. Also, the conversion of glycerol-3-P to glycerol leads to a gain of free phosphate and can hence be considered as a possible reason for the up-regulation of this pathway. Still, a clear impact of reduced phosphate on the glycolytic flux remains to be investigated on a level that was beyond the scope of our study. The determination of activities of the corresponding glycolytic enzymes and the determination of their K_s _values with respect to different phosphate levels could possibly increase the fundamental understanding with respect to the inter-dependency of glycolytic flux and phosphate supply.

The SPA approach identified gene clusters with both decreased (cA1 and cA2) and increased (cB1 and cB2) expression pattern under reduced phosphate supply for the enriched category of *protein folding, modification and transport*. This category was further divided in distinct sub-groups for biological processes which are related to general protein maturation, based on their GO classifications. Four significantly enriched gene sets, i.e. sub-categories that include genes with distinct roles within the protein processing pathway, were identified. We found that genes involved in *ubiquitin-dependent protein catabolism *and in *transport from ER to Golgi *were down-regulated under reduced phosphate supply, whereas genes involved in *cell wall organization and biosynthesis *and in *Golgi vesicle-mediated transport *were up-regulated. These observations suggest that the later secretory processing capacity of the cells (post-Golgi) was enhanced while the retention-and possibly the quality control-of proteins in the ER was reduced when cells were cultures under reduced phosphate concentrations.

It was also found that both the IAP expression under the control of the *TPI1 *promoter and the *TPI1 *transcript levels increased with decreasing phosphate supply. To our knowledge, the transcriptional up-regulation of *TPI1 *as a consequence of reduced phosphate supply has not been reported before. We would like to stress the possibility that the increased IAP transcript levels could also be derived from an increased amplification level of the expression cassette (2 μplasmid) harboring the *IAP *gene, suspecting an increased gene dosage of IAP to cause higher transcript levels. Yet, due to the increase of the transcript level of the genomic *TPI1 *and its similar trend to the *IAP *transcript, we consider *TPI1 *to be transcriptionally regulated by the phosphate supply. Finally, our DEA approach showed a moderate up-regulation of the transcription factor *GCR1 *(1.7 fold), which is known to regulate the high-level expression of *TPI1 *[32]. Although transcription factors are generally believed to be regulated post-transcriptionally, the increased transcription of *GCR1 *is in accordance to our finding regarding the *TPI1 *expression dependency on phosphate availability, and thus can be considered as a possible reason for the up-regulation of *TPI1*. Still, the increased transcription of IAP cannot be considered as the sole reason for the increased secretory production of IAP. The initial decrease of P_i _from [33 mM] to [14.7 mM] did not significantly affect the transcript levels of IAP (increase of 6.9% with a SD of 3.6%), yet the yield of secreted IAP increased 22.6%, suggesting that the global increase of the secretory flux, i.e. the increased secretion of diverse phosphatases and high affinity phosphate transporters, did also benefit the secretory production of IAP. In follow-up studies, and for separately addressing the impact of reduced phosphate supply on both the transcription and the secretion of a heterologous protein, the glycolytic *TPI1 *promoter should be replaced with a non-glycolytic promoter, e.g. *TEF1*, as by de-coupling the two identified conjectures for improved IAP secretion found in this study, more explicit conclusions would be promoted

As *TPI1 *is one of the most commonly used glycolytic promoters for recombinant expression of diverse heterologous proteins in yeast, our data suggests that a special care needs to be taken with the design of the growth media with respect to phosphate supply. For most commonly used media for laboratory scale studies, e.g. derivatives of [33], supply a high amount of phosphate to ensure optimal conditions for biomass formation. The supplied P/C ratio of 0.4 (mM phosphate per mM glucose) is 8 fold higher than the starting P/C ratio we used in this study (0.05). In addition, we report that a two fold gain of cell specific productivity and 25% increase of volumetric productivity can be achieved by further decreasing the phosphate supply to at least half of the initial concentration.

## Conclusions

In the present study, we looked into the physiological impacts of phosphate shortage in an industrial API production process. We identified a positive effect of reduced phosphate supply on the secretory production of human insulin in *S. cerevisiae*. The cellular response towards reduced phosphate levels in the medium was pictured in the up-regulation of multiple phosphatases and permeases that are channeled towards the extracellular space, thus leading to an increased activity of the protein trafficking pathway. By following the successive response of reduced phosphate levels on the physiological state of cells, we identified a range of interest for improved IAP productivity, covering low but not critical P_i _levels. The consecutive methodology we applied for studying the regulatory shifts from cells growing under carbon limitation towards phosphate limitation resulted in more profound insights into the adaptive response of the cells to their nutrient availability. By combining information obtained through the analysis of differentially expressed genes among low and high phosphate levels, with the identification of significant gene expression patterns which followed major phenotypic observations, e.g. IAP productivity, we enlarged the picture of cellular events during growth on reduced phosphate levels.

Our work underlines the inter-connection among the signaling of phosphate scarcity, the re-distribution of glycolytic flux towards glycerol and ethanol production, the phosphatidylinositol biosynthesis, the NAD biosynthesis, and the polyphosphate metabolism. Moreover, we emphasize the importance of optimized media formulation with respect to a reduced P/C ratio, aiming at improved heterologous protein production. We conclude that a dietary supply of phosphate can potentially enhance the secretory production of a heterologous protein. Under reduced phosphate levels, the usage of the glycolytic *TPI1 *promoter further increased the recombinant transcript levels of the IAP gene, advancing the overall productivity of the target protein.

## Methods

### Expression backbone

The haploid *S. cerevisiae *strain CEN.PK113-11C (MAT**a**, *ura3-52 his3-*D*1, MAL2-8*c SUC2) was transformed with the *URA3*d based expression cassette pU17 via LiAc method, according to [34]. The 2 micron vector pU17 is a *HIS3 *plasmid derived from [35], with an expression cassette containing the *TPI1 *promoter and the insulin analogue precursor (IAP) gene that encodes an insulin Asp^B28 ^molecule where the A- and B-chain are connected by a EWK C-peptide [36]. The secretory expression of the insulin precursors was performed with the aid of the mating type factor alpha leader in the configuration: α-factor leader-KR-spacer-insulin precursor, leading to an optimized processing of the fusion protein by the native Kex2 endoprotease, as described by [37]. The expression cassette also contains the *URA3 *open reading frame plus 17 base pairs of the endogenous *URA3 *promoter. Both *HIS3 *and *URA3 *were used as auxotrophic selection markers. The *URA3*d based plasmids provide an increased pool of the recombinant gene (IAP) as the defective *URA3 *gene led to amplified plasmid copy numbers (Kazemi Seresht et al., in preparation).

### High cell density cultivation

The batch phase was followed by a carbon-limited fed-batch phase with an open loop controlled specific growth rate of μ_set _= 0.1 h^-1^. During this phase, 55 gL^-1 ^glucose was added to the process (feed concentration 500 gL^-1^). The fed batch phase was automatically initiated after glucose depletion during the batch phase, monitored and controlled with the decrease of the carbon dioxide signal in the off-gas measurement. The chemostat phase was automatically initiated after the fed batch phase. The culture volume was controlled via a gravimetric feed control, ensuring a fixed dilution rate of D = 0.1 h^-1^. The respiratory quotient (RQ) and the at-line analysis of ethanol in the exhaust gas were monitored, confirming a fully respiratory growth of the cells during the fed batch and chemostat phases, i.e. the chosen dilution rate was lower than the critical dilution rate and no ethanol formation was detected. The pH was controlled at 5.9 by addition of NH_4_OH (10%), which provided a non-limiting nitrogen source throughout the whole process. The temperature was controlled at 28°C, and the dissolved oxygen tension (DOT) was kept above 40% of saturation to avoid oxygen limitation. Cultivations were performed in the Sartorius Stedim Biotech BIOSTAT Q*plus*^® ^(0.4 L scale).

### Media formulation

The defined growth medium for plates was 6.7 gl^-1 ^yeast nitrogen base without amino acids, but with ammonium sulphate, 2% glucose, 2% agar and the relevant CSM drop-out amino acid mixture (from Qbiogene). A defined minimal medium (DMM) was used in all experiments. Its basal composition for pre-cultures (5 mL scale in 50 mL falcon tubes) and shake flasks (100 mL scale in 500 mL shake flasks with two baffles) was: 10.5 gL^-1 ^Potassium hydrogen phthalate (KHP), 5 gL^-1 ^(NH_4_)_2_SO_4_, 2 gL^-1 ^KH_2_PO_4_, 0.5 gL^-1 ^MgSO_4_·7 H_2_O, 0.1 gL^-1 ^CaCl_2_·2 H_2_O, 100 μLL^-1 ^antifoam (Antifoam 204, Sigma-Aldrich), 1 mLL^-1 ^trace metals solution, 1 mLL^-1 ^vitamin stock solution, and an initial glucose concentration of 20 gL^-1^. The trace metal and vitamin solutions were derived from [33]. For chemostat experiments, a modified DMM was used with the following composition: 3 gL^-1 ^KH_2_PO_4_, 2 gL^-1 ^(NH_4_)_2_SO_4_, 1.5 gL^-1 ^MgSO_4_·7 H_2_O, 0.2 gL^-1 ^CaCl_2_·2 H_2_O, 0.1 gL^-1 ^NaCl, 200 μLL^-1 ^antifoam, 10 mLL^-1 ^trace metals solution, 10 mLL^-1 ^vitamin stock solution. The initial glucose concentration in the batch was set to 20 gL^-1^, and in the chemostat medium to 75 gL^-1^. Cultivations were carried out under six different phosphate concentrations, using KH_2_PO_4 _as phosphate source (Table 1). The resulting alternations in the potassium (K) levels were compensated for by addition of KCl.

**Table 1:** The addition of different concentrations of KH_2_PO_4 _and KCl to DMM, for alternating the phosphate levels and maintaining the potassium levels, respectively.

*P_i _conc.* *[culture ID]*	*KH_2_PO_4 _in DMM* *[gL^-1^]*	*KCL in DMM* *[gL^-1^]*
*33 mM*	*4.5*	*-*
***22 mM***	***3.0***	***-***
*14.7 mM*	*2.0*	*0.6*
*9.6 mM*	*1.3*	*1.0*
*5.5 mM*	*0.75*	*1.3*
*2.6 mM*	*0.35*	*1.5*

### Microarray analysis

#### Sampling method

Samples for RNA isolation were taken during the steady state phase of the cultivations. Three samples were withdrawn within 48 hours and used as biological replicates for each culture. For the sampling, 5 mL culture broth were rapidly taken into a 50 mL sterile Falcone tube, prefilled with 25 mL of crushed ice, in order to decrease to sample temperature to below 4°C in a few seconds. Cells were centrifuged (4000 rpm at 0°C for 2 min.), the supernatant discarded, the pellet was frozen in liquid nitrogen and stored at -80°C, and the RNA extraction was performed within a month. Total RNA was extracted using the Ambion RiboPure Yeast Kit (Applied Biosystems, Austin, USA), according to manufacturer's instruction, after thawing the cell pellets on ice. RNA sample integrity was determined with an Agilent 2100 Bioanalyzer and RNA 6000 Nano LabChip kit (Agilent, Santa Clara, CA), ensuring RIN values above 9.0, and total RNA quantity was determined with a NanoDrop 3300 UV-Vis spectrophotometer (Thermo Scientific, Rockford, USA).

#### Microarray analysis and data acquisition

Using the 3'IVT Expression Kit (One-Cycle cDNA Synthesis) and the GeneChip Hybridization, Wash and Stain Kit, the probe preparation and hybridization to Affymetrix Yeast Genome Y2.0 arrays were performed according to manufacturer's instructions (Affymetrix GeneChip Expression Analysis Technical Manual, P/N 702232, Rev.2). Washing and Staining of arrays were performed using the GeneChip Fluidics Station 450 and scanning with the Affymetrix GeneArray 3000 7G Scanner (Affymetrix, Santa Clara, CA). The Affymetrix GeneChip Command Console Software (AGCC) was used to generate CEL files of the scanned arrays. Data analysis was performed using the statistical open source language R (R Development Core Team, http://www.r-project.org), supplemented with Bioconductor v2.8 (Bioconductor Core Team, http://www.bioconductor.org). Data preprocessing was carried out by using the Robust Multichip Average method, considering the calculated probe affinity derived from position dependent base effects (GC-RMA), including a quantile normalization of probe intensities and a median polishing step for expression measure calculation.

#### Gene Expression Analysis

For the Differential Expression Analysis (DEA), the Linear Models for Microarray Data (LIMMA) method was applied, using the limma statistical package. A moderated *t*-test between the two experiment sets of low (9.6 mM P_i_) and high (22 mM P_i_) phosphate supplemented cultures was used for pair-wise comparison. Empirical Bayesian statistics were used to moderate the standard errors within each gene and Benjamini-Hochberg's method was applied to adjust for multiple testing. A cut-off value of adjusted *p *< 0.05 was chosen for statistical significance. The statistically significant differential gene expression list was further trimmed by considering only genes with |fold change| > 2 as biological significant. Significant gene ontology (GO) process terms of the selected genes were identified using GO Slim Mapper tools (Saccharomyces Genome Database SGD) with a threshold of *p *< 0.01.

For the Significant Profile Analysis (SPA), the MicroArray Significant Profiles (maSigPro) method was applied, using the masigpro statistical package. A two-step linear regression model was used to identify genes with a significant expression profile as a response to alternating phosphate supply, considering gene sets of five different cultures growing at 5.5 mM, 9.6 mM, 14.7 mM, 22 mM, and 33 mM phosphate concentration in the feed reservoir. The step-wise regression was adjusted to polynomial degree of 2, a significance cut-off of α = 0.05 was used, and an R-squared cut-off of 0.7 was chosen to increase the goodness of the fit. The identified significant gene set was clustered in 10 subsets using a hierarchical clustering method. A similar GO enrichment analysis as for the DEA approach was applied to the identified gene subsets

### Analytical methods

#### Extracellular metabolites

The extra-cellular metabolites glucose, glycerol, acetate, and ethanol were analyzed on a reverse phase HPLC (Agilent 1200), separated on a Rezex ROA-organic acid H^+ ^(8%) 7.8 × 300 mm column (Phenomenex), operating at 45°C and at a flow rate of 0.5 mLmin^-1^, using a 5 mM H_2_SO_4 _solution as running buffer. Detection was done using a refractive index detector.

#### Human insulin

Insulin was separated on a reverse phase HPLC (Agilent 1200) on a C4 Jupiter 4.6 × 250 mm (5 μm) column (Phenomenex), operating at 42°C and at a flow rate of 1 mLmin^-1^. Buffer A was composed of 0.1% TFA in milliQ water, and buffer B was composed of 0.07% TFA in acetonitrile, with an elution gradient of 20-80% B in 20 minutes, 100% B in 5 minutes, 100-20% B in 0.1 minutes. The UV-detection was performed at 214 nm, and the concentration was determined using Human Insulin as external standard.

#### RT-qPCR

Total RNA was extracted using the Ambion RiboPure Yeast Kit (Applied Biosystems, Austin, USA), according to manufacturer's instructions. The RNA extracts were treated with DNAse (TURBO DNAse, Ambion), and the RNA concentration was measured with a Spectrophotometer (Nanodrop 3300^®^, Thermo Scientific). For cDNA synthesis, the High Capacity cDNA Reverse Transcription Kit (Applied Biosystems) was used. Quantification of the cDNA levels of the insulin gene (IAP), *ACT1 *and *TPI1 *was performed by quantitative PCR using a Stratagene MX3005p^® ^instrument. *ACT1 *was used as reference gene, with the primer set 5'-GCCTTCTACGTTTCCATCCA-3' and 5'-GGCCAAATCGATTCTCAAAA-3'. Transcript levels were determined for the IAP gene, using the primer set 5'-GAAGCTGAAGCTCCAAAGTTCG-3' and 5'-CGGGCTGCGTCTAGTTACAGTAG-3', and for *TPI1*, using the primer set 5'-TCCCAGAAAATGTCGAAGTTG-3' and 5'-GGCTTCTTAACCAAAGAGACAGA-3'. Standard curves were generated pooled cDNA from all samples, and efficiencies close to 100% were achieved (E ≥ 95%). Potential primer dimerization effects were excluded via melting curve analysis.

### Calculations of yields and productivities

All yield and productivity calculations are based on averaged concentrations of the corresponding compound, derived from samples that were taken during the steady state phase of cultivations. The biomass yield is represented as y_X/S _and based on the mass of consumed glucose (g/g), whereas the yield of the metabolites ethanol and glycerol are represented as Y_E/S _and Y_G/S _and based on the molar mass of consumed glucose (mmol/mol), respectively. The insulin production yield y_IAP/X _is based on the cell dry weight (mg/g). The specific insulin productivity q_IAP/X _is defined as the product of the dilution rate (D) and insulin production yield (y_IAP/X_) during the steady state phase of chemostat cultivations.

## Competing interests

The authors declare that they have no competing interests.

## Authors' contributions

LO and EAP supervised the research and contributed to the revision of the manuscript. AKS designed and performed the experiments, carried out the data analysis and drafted the manuscript. All authors have read and approved the manuscript.

## Supplementary Material

Additional file 1 **Sum-up of the GO Slim enrichment analysis**. A detailed illustration of the GO Slim enriched categories of clusters cA1 (page 1), cA2 (page 2), cB1 (page 3), and cB2 (page 4) is shown as presented in Figure 3. Click here for file
