# Supplementary material for: The impact of phosphate scarcity on pharmaceutical protein production in S. cerevisiae: linking transcriptomic insights to phenotypic responses
Source: Microb Cell Fact. 2011 Dec 7;10:104. doi: 10.1186/1475-2859-10-104 (PMC3265430; doi:10.1186/1475-2859-10-104)
Supplement: Additional file 1 — Sum-up of the GO Slim enrichment analysis. A detailed illustration of the GO Slim enriched categories of clusters cA1 (page 1), cA2 (page 2), cB1 (page 3), and cB2 (page 4) is shown as presented in Figure 3. [file 1475-2859-10-104-S1.PDF]

Supplementary file

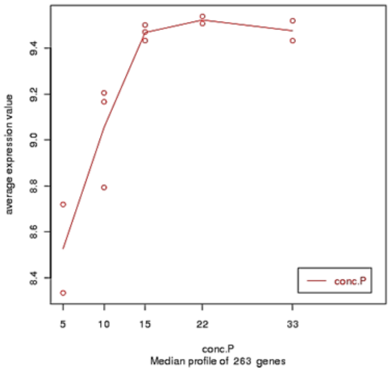

Cluster: **cA1**

Genes: ***263 genes***

Selected enriched Categories:

Category	GO Slim sub-categories	Frequency	Genome Frequency	*Gene(s)*
transport		***19.8 %***		
	transport	52 out of 263 genes, 19.8%	1048 of 6311 genes, 16.6%	*SSA1,SEC18,RER1,FEN2,THR4,KIN82, YDL199C,SSB1,RLI1,TVP15,SEC1,RTN1,MTH, HXT7,STL1,YCK3,EMP47,YFL054C,HXK1, PEX14,ATG1,MDM34,HXK2,GOS1,MIP6, NMD3,SEC28,TPM2,SSC1,SFC1,ENT3,SSH4 JEN1,VPS1,PAM17,SRP40,HSP60,CHS5,VTI1, TOM22,MEP2,ACC1,SOL1,MSO1,TIM18, SNC2,PUT4,PMA2,VPS28,ODC1,SRP72,FLC1*
protein folding, modification, transport		***15.6 %***		
	protein modification process	17 out of 263 genes, 6.5%	590 of 6311 genes, 9.3%	*ACS1,RFA1,PAF1,KIN82,UBC5, GIS1,SNF1, GIP2,MOT2,YCK3,ATG1,SIP2,CTM1,ELP6, PFA3, CYC2,PNG1*
	protein complex biogenesis	13 out of 263 genes, 4.9%	210 of 6311 genes, 3.3%	*UMP1,PET117,PRE4,PEX14,SIP2, SHY1,SSP1, NAS2,HSP60,EIS1, ATG34,CYC2,SRP72*
	cellular protein catabolic process	10 out of 263 genes, 3.8%	198 of 6311 genes, 3.1%	*UMP1,MFB1,PRE1,MDJ1,PRE4,NAS2,DAS1, SSH4,VPS28,PNG1*
	protein folding	9 out of 263 genes, 3.4%	88 of 6311 genes, 1.4%	*SSA1,SSB1,MDJ1,SSC1,HSP60,GSF2,SIS1,STI1 FLC1*
cellular membrane organization		***11 %***		
	cellular membrane organization	21 out of 263 genes, 8%	286 of 6311 genes, 4.5%	*SEC18,FEN2,THR4,KIN82,SEC1,ATG1,GOS1 SEC28,ENT3,VPS1,MEH1,FCJ1,VTI1,TOM22,P FA3,ACC1,MSO1,CYC2,SEY1,TIM18,SNC2*
	vesicle-mediated transport	18 out of 263 genes, 6.8%	366 of 6311 genes, 5.8%	*SEC18,RER1,FEN2,THR4,TVP15,SEC1,YCK3, EMP47,GOS1,SEC28,TPM2,ENT3,SSH4,VPS1, CHS5,VTI1,MSO1,SNC2*
	vacuole organization	8 out of 263 genes, 3.0%	130 of 6311 genes, 2.1%	*SEC18,ATG1,TPM2,SSH4,VPS1,VTI1,PFA3, VPS28*
	vesicle organization	6 out of 263 genes, 2.3%	77 of 6311 genes, 1.2%	*SEC18,SEC1,GOS1,SEC28,VTI1,SNC2*
biological process unknown		***19.8%***		
	biological process unknown	52 out of 263 genes, 19.8%	1214 of 6311 genes, 19.2%	*RTC2,OM14,YCP4,TMA17,YDL121C,YDR090C AIM9,AIM11,FMP32,RRT6,YRB30,NQM1, HGH1,CIR1,YGR235C,YHR045W,YHR097C, YHR131C,YHR192W,YHR202W,YIL024C, OM45,YIR016W,MTC1,NIT2,YJL160C, YJR008W,YKL091C,YKL100C,FMP46,YLR001C YLR036C,YLR149C,YLR312C,TMA10,ART10, YLR446W,YML053C,SPG4,SPG5,YMR196W, AIM37,YNL181W,YNL195C,YTP1,YNR021W, YNR034W-A,AIM39,CUE5,AIM41,UIP4, YPL229W*

Supplementary file

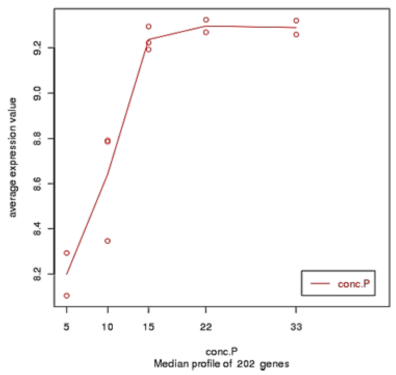

Cluster: **cA2**

Genes: ***202 genes***

Selected enriched Categories:

Category	GO Slim sub-categories	Frequency	Genome Frequency	*Gene(s)*
transport		***19.3 %***		
	transport	39 out of 202 genes, 19.3%	1048 of 6311 genes, 16.6%	*MDM10,SSA3,BAP2,MAL31,ADY2,MCH1, BUG1,ATG9,ENT1,YDL183C,SNF3,GGC1, TMN2,PEX7,PEX5,TRS23,ATO3,UGO1,GDI1, DDI1,FLC3,MAL11,YCK1,PEX18,SEC11,PEP8, OCT1,PXA2,MIA40,VTA1,ATG23,ESBP6,SRP1, SHR5,FRE7,UFE1,PXA1,SRO7,ANT1*
protein folding, modification, transport		***19.8 %***		
	protein modification process	15 out of 202 genes, 7.4%	590 of 6311 genes, 9.3%	*MRK1,PPH3,NBP2,EPL1,YCK1,YUH1,UBA1, GPI13,CDC73,YPK2,LAG2,PKH2,SHR5,ALG8, TAF14*
	protein complex biogenesis	10 out of 202 genes, 5%	210 of 6311 genes, 3.3%	*MDM10,ATG9,PEX7,PEX5,VMA22,COX23, DNM1,VTA1,YPK2,HSC82*
	vesicle-mediated transport	10 out of 202 genes, 5%	366 of 6311 genes, 5.8%	*BUG1,ENT1,TRS23,GDI1,DDI1,YCK1,PEP8, VTA1,UFE1,SRO7*
	cellular protein catabolic process	5 out of 202 genes, 2.5%	198 of 6311 genes, 3.1%	*SAF1,DDI1,EPS1,CPS1,CDC31*
	protein folding	5 out of 202 genes, 2.5%	88 of 6311 genes, 1.4%	*SSA3,CAJ1,CPR6,HSC82,MPD2*
response to stress and chemical stimulus		***13.4 %***		
	response to stress	20 out of 202 genes, 9.9%	606 of 6311 genes, 9.6%	SSA3,ZTA1,MRK1,ATG9,SNQ2,PPH3,SHU2, NBP2,CTA1,SVF1,MAG1,EPL1,OPI1,RRD1, OSM1,SFH1,ATG23,TPP1,HSC82,ATH1
	response to chemical stimulus	12 out of 202 genes, 5.9%	334 of 6311 genes, 5.3%	ZTA1,SNF3,GAL3,SNQ2,CTA1,SVF1,IZH1,SLI1, OPI1,YCK1,OSM1,ASC1
biological process unknown		***21.8%***		
	biological process unknown	44 out of 202 genes, 21.8%	1214 of 6311 genes, 19.2%	*UBP13,YBR053C,YBR062C,TBS1,RNQ1,PEX34 SNA4,YDL157C,OCA6,DOS2,ALT2,YDR186C, CNL1,YEL057C,HVG1,YER039C-A,YGL007C-A,YGL010W,FMP37,YGL081W,AIM14,IMO32 YGR067C,YGR130C,SPG1,FMP43,YGR250C, SET5,TDA4,YKR075C,PCD1,YMR147W,OSW, YMR206W,APJ1,GPM3,YOR238W,RDL1, YOR352W,CIR2,MGR2,AIM43,YPL109C, PRM4*

Supplementary file

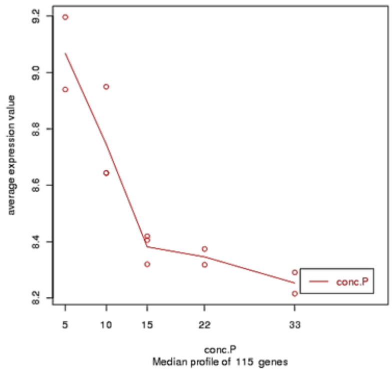

Cluster: **cB1**

Genes: ***115 genes***

Selected enriched Categories:

Category	GO Slim sub-categories	Frequency	Genome Frequency	*Gene(s)*
transport		***20 %***		
	transport	23 out of 115 genes, 20%	1048 of 6311 genes, 16.6%	*SYN8, PEP1, DNF2, PEP7, PAC11, PMP2, GET2, DNF1, ERP5, LOT6, ZRT2, EAR1, MSG5, MSK1, AVT4, PHO91, SIA1, MEX67, POR2, FRE2, DYN1, NEW1, RGC1*
protein folding, modification, transport		***23.5 %***		
	protein modification process	14 out of 115 genes, 12.2%	587 of 6311 genes, 9.3%	*SWD1,UBC4,PPS1,PMT5,SMT3,CWH41,NCS6 PMT6,HOG1,TAF9,MSG5,MNT4,HRT1,KIN4*
	protein complex biogenesis	2 out of 115 genes, 1.7%	210 of 6311 genes, 3.3%	*PAC10,PKR1*
	cellular protein catabolic process	6 out of 115 genes, 5.2%	198 of 6311 genes, 3.1%	*PEP1,UBC4,YLR224W,UFO1,EAR1,HRT1*
	vesicle-mediated transport	6 out of 115 genes, 5.2%	366 of 6311 genes, 5.8%	*DNF2,PEP7,GET2,DNF1,ERP5,EAR1*
	vesicle organization	3 out of 115 genes, 2.6%	77 of 6311 genes, 1.2%	*PEP7, OYE2,KEX2*
biological process unknown		***19.8%***		
	biological process unknown	25 out of 115 genes, 21.7%	1214 of 6311 genes, 19.2%	*PBY1,YDR061W,AMD2,YDR541C,YER152C YHR078W,YHR214C-D, UBP12,MTC2, YKR043C,YLR177W,YLR179C,YLR225C,NIT3, MAG2,YMR209C,GAS3,YMR259C,YNL035C, YNL046W,YNR029C,YOR032W-A, YOR246C, YPL168W, YPL199C*

Supplementary file

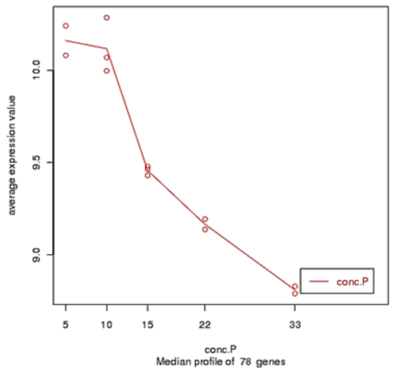

Cluster: **cB2**

Genes: ***78 genes***

Selected enriched Categories:

Category	GO Slim sub-categories	Frequency	Genome Frequency	*Gene(s)*
transport		***19.1 %***		
	transport	15 out of 78 genes, 19.1%	1048 of 6311 genes, 16.6%	*RFT1,PHO89,ADP1,GIT1,VTC1,VTC2,OSH7,GPA1,GGA2,VTC4,PHO86,STE6,PHO84,ALR1,VTC3*
protein folding, modification, transport		***19.2 %***		
	protein modification process	12 out of 78 genes, 15.4%	587 of 6311 genes, 9.3%	*CYC3,RFT1,MNN2,ALG7,SAT4,PHO13,PHO8,KRE2,MNN1,CLB1,ELM1,KTR2*
	protein complex biogenesis	1 out of 78 genes, 1.3%	210 of 6311 genes, 3.3%	*CYC3*
	vesicle-mediated transport	3 out of 78 genes, 3.8%	366 of 6311 genes, 5.8%	*OSH7,GGA2,PHO86*
	protein folding	1 out of 78 genes, 1.3%	88 of 6311 genes, 1.4%	*PHO86*
cellular carbohydrate metabolic process		***15.4 %***		
	cellular carbohydrate metabolic process	12 out of 78 genes, 15.4%	265 of 6311 genes, 4.2%	*CDC19,RFT1,MNN2,ALG7,KRE2,MNN1,HOR2 ELM1,KTR2,PRS5,ADH1,DDP1*
biological process unknown		***20.5 %***		
	biological process unknown	16 out of 78 genes, 20.5%	1214 of 6311 genes, 19.2%	*YPK3,YBR287W,GFD2,PHM6,GTT3,HMF1,YHR033W,YHR210C,YIL169C,YLR063W,YLR346C,YNL217W,ZPS1,YOL155W-A,YOR342C,YOR390W*
